# Rapid Estimation of Gustatory Sensitivity Thresholds with SIAM and QUEST

**DOI:** 10.3389/fpsyg.2017.00981

**Published:** 2017-06-16

**Authors:** Richard Höchenberger, Kathrin Ohla

**Affiliations:** ^1^Psychophysiology of Food Perception, German Institute of Human Nutrition Potsdam-RehbrueckeNuthetal, Germany; ^2^NutriAct – Competence Cluster Nutrition Research Berlin-PotsdamNuthetal, Germany

**Keywords:** taste sensitivity, threshold, gustation, SIAM, QUEST

## Abstract

Adaptive methods provide quick and reliable estimates of sensory sensitivity. Yet, these procedures are typically developed for and applied to the non-chemical senses only, i.e., to vision, audition, and somatosensation. The relatively long inter-stimulus-intervals in gustatory studies, which are required to minimize adaptation and habituation, call for time-efficient threshold estimations. We therefore tested the suitability of two adaptive yes-no methods based on SIAM and QUEST for rapid estimation of taste sensitivity by comparing test-retest reliability for sucrose, citric acid, sodium chloride, and quinine hydrochloride thresholds. We show that taste thresholds can be obtained in a time efficient manner with both methods (within only 6.5 min on average using QUEST and ~9.5 min using SIAM). QUEST yielded higher test-retest correlations than SIAM in three of the four tastants. Either method allows for taste threshold estimation with low strain on participants, rendering them particularly advantageous for use in subjects with limited attentional or mnemonic capacities, and for time-constrained applications during cohort studies or in the testing of patients and children.

## 1. Introduction

The ability to taste enables detection of nutrients and toxins in the oral cavity and is therefore a crucial determinant for decisions as to whether to consume or reject a food. Taste function is typically viewed as the ability to detect or discriminate unique taste qualities (salty, sour, sweet, bitter, and umami). The assessment of taste function is pertinent to identify selective (for one taste) or generalized (for all tastes) taste impairment, as these may lead to deviant eating behavior and, hence, over- or malnutrition.

Sensory sensitivity is a good measure of the overall function of a sensory system. It refers to the ability to detect or identify a particular stimulus and can be quantified by estimating the *threshold intensity*, i.e., the concentration of a stimulus which can be perceived with a certain proportion on repetitive exposures. A common procedure to measure thresholds is the method of constant stimuli (MCS), which samples the complete perceptual range, allowing for the assessment of the entire psychometric function (PF). It requires the time-consuming repeated presentation of stimuli over a wide intensity range. Adaptive methods, on the other hand, either implicitly focus on a specific threshold (e.g., the simple up/down staircase), or make explicit assumptions about an underlying PF and try to recover the parameters governing its shape (e.g., Bayesian procedures like QUEST). Adaptive methods are particularly advantageous as they can help to markedly reduce the number of trials and the overall testing time.

In any threshold estimation method, participants perform a specific *task* to produce a response. Forced-choice paradigms are typically considered to produce a criterion-free, unbiased threshold estimate (Green and Swets, 1966). For example, in a 2-AFC task participants are simultaneously presented with two stimuli per trial (*signal* + *noise* and *noise only*), and the task is to identify the signal. While simultaneous stimulus presentation can be easily achieved with non-chemical stimuli, e.g., on a split screen in vision or binaural stimulation in audition, two different liquid stimuli cannot be applied to the tongue at the same time while maintaining their individual stimulus properties; instead, their mixture would be perceived as a single stimulus. Therefore, taste stimuli are typically applied in sequential order, e.g., in a *temporal 2-AFC* or *two-interval forced-choice* (2-IFC) task. Here, the stimuli are presented in two so-called *intervals* and participants are required to select the interval which contained the signal. The 2-IFC design can be extended to more than two stimuli; in fact, the 3-IFC (with three stimulus presentations per trial) and the related triangle test, in which the “odd” stimulus is to be selected, are frequently applied for gustatory threshold estimation and discrimination testing (O'Mahony, 1995).

IFC tasks impose a strain on participants' memory, as they are required to correctly memorize, recall, and retrospectively compare and judge two or more stimuli. What may only be a minor problem in audition, touch or vision, where stimuli can be presented in rapid succession, poses a difficulty in gustation because this sense is particularly prone to sensory adaptation and habituation. Accordingly, gustatory inter-stimulus intervals (ISI) typically last between 15 and 30 s. However, it has been suggested that long ISIs prevent participants from directly comparing the stimuli (Kaernbach, 1990). Further, participants may “prefer” one interval over the other, a phenomenon called *interval bias*, potentially confounding the procedure (Klein, 2001). Moreover, AFC and IFC tasks have been found to be less efficient in terms of information gain per trial, compared to so-called *yes-no tasks* (e.g., Rose et al., 1970; Kershaw, 1985; Madigan and Williams, 1987).

In a *yes-no* paradigm, participants are presented with only a single stimulus per trial and have to respond whether or not they perceived it (either *Yes* or *No*). Adaptive yes-no tasks provide an efficient tool for threshold estimation as they minimize redundant stimulus presentations (and thereby testing time) while still providing accurate results (Kaernbach, 1990; King-Smith et al., 1994). Naturally, these procedures do not introduce an interval bias, and are inherently intuitive to the participant. However, to date there have only been few applications of the yes-no task in the gustatory domain (e.g., Hautus et al., 2010; Ohla et al., 2010; Shepherd et al., 2011).

Kaernbach's single-interval adjustment-matrix (SIAM; Kaernbach, 1990) algorithm allows for a bias-free threshold estimation in a yes-no design. Participants are presented with *target stimuli* and *blanks* in random order, and have to state whether or not they received the target. If a target is correctly recognized, the response is considered a *hit*; otherwise, it's a *miss*. If a blank is reported as such, the response is considered a *correct rejection*; if, however, it is mistaken for a target, the response is a *false alarm*. Depending on the type of response, the stimulus intensity level for the next trial is adjusted: for a hit, intensity is decreased; for a miss and a false alarm, it is increased; for a correct rejection, it remains unchanged. The numbers of respective steps are summarized in an *adjustment matrix*. To our knowledge, SIAM has been used in taste research in only two published studies so far: both Hautus et al. (2010) and Shepherd et al. (2011) estimated sucrose thresholds in paradigms with 30 and 60 trials per session, respectively. The bias-free approach SIAM is taking comes at a price: typically, half of trials contain blanks, effectively reducing the number of taste stimuli to 50% for a fixed set of trials.

Some adaptive methods follow a maximum-likelihood (ML) approach to select the next stimulus intensity based on all previous responses. The goal is to place the stimulus such that the anticipated information gain about the true threshold will be maximized. ML procedures are known to “converge […] very quickly and make good use of all the data,” and should therefore be preferred “when testing must be accomplished very quickly, as in testing animals or infants” (Leek, 2001). Linschoten et al. (2001) used Harvey's (1997) ML-PEST to measure gustatory and olfactory thresholds in a 2-IFC design. The related QUEST procedure (Watson and Pelli, 1983) follows a Bayesian approach and allows for the specification of prior assumptions (i.e., probability distribution of the true threshold), which can further improve efficiency. Its suitability for the measurement of taste sensitivity thresholds has only recently been demonstrated (Hardikar et al., 2016).

Any threshold estimation method will require substantially more time in gustation than in the non-chemical senses, where ISIs can be much shorter. Since long experiments pose cognitive strain on participants, thereby potentially compromising performance of the procedure, the need for efficient, yet reliable tools is evident. In an attempt to reduce the overall testing time, we evaluated the applicability and test-retest reliability of slightly modified SIAM and QUEST algorithms for the assessment of taste sensitivity.

## 2. Methods

### 2.1. Participants

Forty two healthy participants (median age: 29 yr, range: 20 yr to 65 yr; median BMI: 23.1 kg m-2, range: 18.0 kg m-2 to 39.0 kg m-2; median hip-waist ratio: 1.20, range: 0.76–1.44; 24 women) were recruited and received compensatory payment for their participation. Exclusion criteria were: self-reported taste and smell disorders, smoking, current or recent oral, nasal or sinus infections, pregnancy, recent (during the last 6 months) childbirth, thyroid disorders, diabetes, and weight loss exceeding 10 kg in the last 3 months. All participants gave written informed consent prior to the experiment. The study was approved by the ethics board of the German Psychological Society (DGPs).

### 2.2. Stimuli

Tastants were sucrose (sweet; *M* = 342.30 g mol^−1^; Sigma-Aldrich, CAS Number: 57-50-1), citric acid (sour; *M* = 192.12 g mol^−1^; Sigma-Aldrich, CAS Number: 77-92-9); sodium chloride (salty; *M* = 58.44 g mol^−1^; Sigma-Aldrich, CAS Number: 7647-14-5), and quinine hydrochloride (bitter; *M* = 396.91 g mol^−1^; Sigma-Aldrich, CAS Number: 6119-47-7) diluted in deionized (DI) water. Based on pilot testing, we prepared sets of different concentrations for each taste quality individually. Concentration steps were equidistantly spaced on a decadic logarithmic grid as follows: sour, 0.015 mm to 46.846 mm (14 log_10_ steps; step width: 0.269); salty, 0.342 mm to 342.231 mm (12 log_10_ steps; step width: 0.273); sweet, 0.073 mm to 584.283 mm (14 log_10_ steps; step width: 0.300); bitter, 0.383 × 10-3 mm to 3.131 mm (18 log_10_ steps; step width: 0.230); see Table 1 for a complete list. DI water was used as a *blank* stimulus.

**Table 1:** Stimulus concentrations used in this study.

**Concentration in mm**				
**Sample**	**Sucrose**	**Citric acid**	**Sodium chloride**	**Quinine hydrochloride**
1	584.282793	46.845721	342.231348	3.130661
2	292.772422	25.240735	182.638604	1.842925
3	146.701724	13.599885	97.468686^*^	1.084860
4	73.508618^*^	7.327712^*^	52.016085	0.638619
5	36.833187	3.948209	27.759411	0.375942
6	18.457493	2.127316	14.814339	0.221294
7	9.249197	1.146211	7.906057	0.130278
8	4.633363	0.617583	4.219199	0.076685^*^
9	2.322524	0.332761	2.251711	0.045138
10	1.162723	0.179315	1.201574	0.026567
11	0.584283	0.096606	0.641342	0.015633
12	0.290885	0.052051	0.342231	0.009196
13	0.146392	0.028055		0.005415
14	0.073357	0.015095		0.003193
15				0.001890
16				0.001098
17				0.000639
18				0.000383

*Starting concentrations are marked with an asterisk*.

All tastants and the water were transferred to small glass bottles equipped with a spray head, and stored refrigerated at 5°C for a maximum duration of 7 days. Taste stimuli were aliquots of ~0.2 mL each, delivered to the anterior third (i.e., the tip) of the tongue.

### 2.3. Apparatus

Stimulus presentation was guided by a Python computer program based on PsychoPy 1.83.03 (Peirce, 2009) running on Windows 7 (Microsoft Corp., Redmond, WA). It displayed written directions on the computer screen, instructing the experimenter which stimulus to present next. Stimulus selection was based on the algorithms described below. A computer keyboard was used to record participants' responses. The source code will be provided upon request.

### 2.4. Procedure

#### 2.4.1. SIAM-based algorithm

The payoff matrix used was based on the one proposed by Kaernbach (1990) for a *target performance* of *t* = 0.5, which translates into a proportion of 75% *Yes* responses (see Table 2). To advance to the threshold concentration more quickly at the beginning of the testing session, we changed the number of adjustment steps in case of a hit from 1 to 3 until the first miss or false alarm was observed. We then switched back Kaernbach's original matrix. Tastants and blanks were presented in random order. 50% of the presented stimuli were blanks. No more than two blanks were delivered in succession. The procedure finished after 30 trials. Due to a technical difficulty, it finished after only 20 trials for two participants. We will refer to this method as “SIAM” in the following.

**Table 2:** Single-interval adjustment matrix: the number of steps to move up or down on the concentration scale based on the previous response.

**Stimulus**	**Response**	
	**Yes**	**No**
Tastant	−1^*^	1
Water	2	0

* *−3 until the first miss or false alarm*.

#### 2.4.2. QUEST-based algorithm

Following Watson and Pelli (1983), the QUEST procedure assumed a Weibull-shaped PF with a slope parameter β = 3.5. Both, lower asymptote (representing false alarm rate) and lapse rate were assumed to be virtually zero, and consequently set to 0.01. Therefore, the PF expanded over the interval [0.01, 0.99] proportion of *Yes* responses. Prior estimates of the true thresholds were relatively flat normal distributions with a standard deviation of 20, centered on the respective starting concentrations of each taste quality. The target threshold was set to 80% *Yes* responses, which causes the algorithm to operate at the ideal sweat factor (Watson and Pelli, 1983) for maximum efficiency. The granularity of the concentration grid was set to 0.01.

QUEST proposes stimulus intensities that will maximize the information gain about the true threshold. These proposals were calculated based on the “best” quantile of the latest estimated psychometric function; this is the default behavior for PsychoPy's QUEST implementation. Because QUEST's internal grid was much more fine-grained than the set of stimuli prepared, we designed the algorithm to select the concentration that was closest to the one proposed by the algorithm. We implemented special handling for the case that a stimulus had already been presented in the previous trial, to avoid repeated presentation of the exact same stimulus concentration: If the participant had recognized the stimulus, we moved one concentration step down for the current trial; if however the participant had failed to recognize the stimulus, we moved one concentration step up. The procedure required the completion of at least 10 and no more than 20 trials, and finished before the 20th trial if the 5 % to 95 % confidence interval was smaller than half the concentration presented last. This method will be referred to as “QUEST” throughout the manuscript.

#### 2.4.3. Threshold estimation

Participants were invited to four separate testing session during which recognition thresholds for the four basic taste qualities sour, salty, sweet, and bitter were measured using either the SIAM or QUEST method. Participants completed both test and retest for the same method in subsequent testing sessions; i.e., methods were never interleaved. The orders of methods and taste qualities within a session were counterbalanced across participants. Within a given method and for a given participant, the order of taste qualities presented in the first (test) and second (retest) testing sessions was identical. The average time between sessions was 1.4 d (range: 1 d to 5 d) for SIAM and 1.6 d (range: 1 d to 6 d) for QUEST. Testing sessions took place at approximately the same time of the day.

Participants were seated in a chair, blindfolded, and instructed which taste quality to attend to. At the beginning of each trial, participants were to stick out their tongue and the experimenter sprayed the stimulus (tastant or blank for SIAM, only tastant for QUEST) onto the anterior portion of the tongue. With the mouth still opened, participants judged whether or not the announced taste was present, and provide a prompt verbal response (*Yes* or *No*). To enforce a conservative decision criterion, they were instructed to respond *Yes* only when certain, and *No* otherwise. If they failed to respond promptly, the experimenter urged them to give a response and, in case of hesitation, logged a *No* response.

During the SIAM procedure, participants received immediate feedback as to whether the presented stimulus was indeed a tastant or a blank. (*This was {sweet, sour, salty, bitter}*. or *This was water*.) Terms like *correct* and *incorrect* were avoided to not communicate any implicit evaluation, which might have confounded the threshold estimation. No feedback was provided during the QUEST procedure.

The experimenter entered the response into the computer, while participants rinsed their mouth with DI water (volume as desired). Neither stimulus nor rinsing water were swallowed, but disgorged into a plastic bowl.

Stimuli were presented with an ISI of 20 s (SIAM) or 30 s (QUEST). The starting concentrations were selected to be clearly perceptible by most participants (based on pilot testing): 7.328 mm for citric acid, 97.469 mm for sodium chloride, 73.509 mm for sucrose, and 0.077 mm for quinine hydrochloride (see also Table 1). After the algorithm finished according to the stopping rules described in 2.4.1 and 2.4.2, testing proceeded with the next taste quality. A total of 672 threshold estimations was completed (42 participants × 4 taste qualities × 2 methods × 2 sessions). Because QUEST's target performance was slightly higher than SIAM's, we expected higher threshold estimates for QUEST compared to SIAM.

### 2.5. Analysis

#### 2.5.1. Threshold estimation and preprocessing

To obtain threshold estimates for each individual SIAM session, we removed the first reversal point if the number of reversals was odd, or the first two reversals if the number of reversals was even. We then calculated the threshold as the arithmetic mean of the remaining reversal concentrations. For QUEST, we retrieved the means of the posterior PDFs, as the mean is thought to be the most efficient and unbiased estimator (King-Smith et al., 1994).

For SIAM, we excluded 21 thresholds because participants either responded *Yes* to the lowest or *No* to highest concentration, three thresholds because no miss was observed, and six thresholds because of erratic responses during the session. Similarly, 19 QUEST thresholds were excluded because participants either responded *Yes* to the lowest concentration, suggesting that the range of tastant concentrations was insufficient for these participants.

To assess whether the selected range of stimulus concentrations was sufficient, we created histograms of threshold estimates as a function of dilution steps. We expected only few (or no) thresholds at the lowest and highest concentrations, respectively.

We obtained mean thresholds by averaging thresholds from test and retest within participants for each taste quality. If either during test or retest a threshold could not be estimated for a participant, the corresponding threshold from the other session was excluded from analysis as well. Accordingly, 42 SIAM (14.3%) and 22 QUEST (6.5%) thresholds were removed, leaving 608 threshold estimates for analysis.

#### 2.5.2. Statistical analysis

To estimate the quality and reliability of threshold estimates, we compared mean thresholds and correlation coefficients between test and retest.

We fitted separate linear mixed models (LMM) for each taste quality using the R package lme4 1.1-12 (Bates et al., 2015) to allow for comparisons of groups with unequal sizes. Fixed effects were method (SIAM, QUEST) and session (test, retest), and their interactions. We included a random intercept for each participant. *p*-values were estimated via afex 0.16-1 (https://github.com/singmann/afex). Degrees of freedom were derived by Kenward-Roger approximation. For pairwise *post-hoc* comparisons, we calculated least-squares means and their contrasts via lsmeans 2.25 (Lenth, 2016), and derived *t*-statistics using multcomp 1.4-6 (Hothorn et al., 2008). *p*-values were Holm-Bonferroni-corrected for multiple testing.

To test for a linear relationship between test and retest threshold estimates, we calculated Pearson's correlation coefficients, *r*, for each taste quality and staircase method individually. Further, we computed correlations between SIAM and QUEST thresholds in order to assess correspondence between both methods. The correlations were calculated using SciPy 0.18.1 (https://www.scipy.org/).

## 3. Results

### 3.1. Staircases

Since the number of trials was fixed *a-priori* in SIAM, threshold estimation took ~9.5 min for all participants. Participants responded *Yes* on 42% of all trials. On average, they produced 10.2 hits (68%), 4.7 misses (31%), 2.3 false alarms (15%), 12.6 correct rejections (84%), and 9.4 reversals. QUEST threshold estimation was completed after 14 trials on average (range: 12–20 trials), corresponding to a duration of 6.5 min (range: 5.5 min to 9.5 min), and finished before the 20th trial in more than 97% of all experiments. Participants responded *Yes* on 63% of all trials. Overall, QUEST seemed to converge faster to the stimulus region and was more robust to erratic responses than SIAM in most experiments. Specifically, SIAM sometimes required numerous trials to recover from false alarms early in a session, which reduced the number of observable reversals and, consequently, the quality of the threshold estimate in such a situation.

### 3.2. Concentration ranges

**Figure 1:**
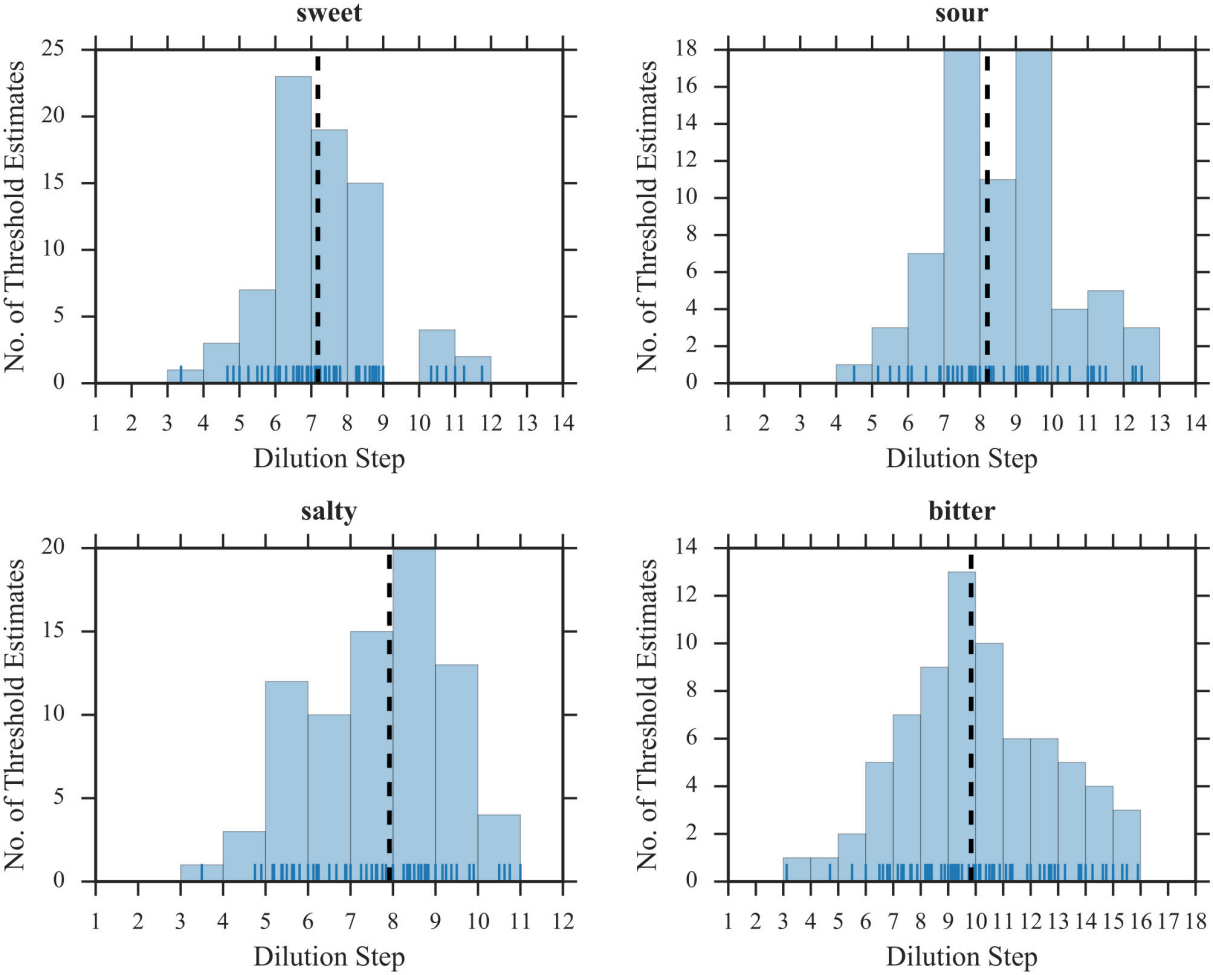
Distribution of threshold estimates for SIAM. Blue vertical lines depict individual participants; black dashed lines represent the medians.

**Figure 2:**
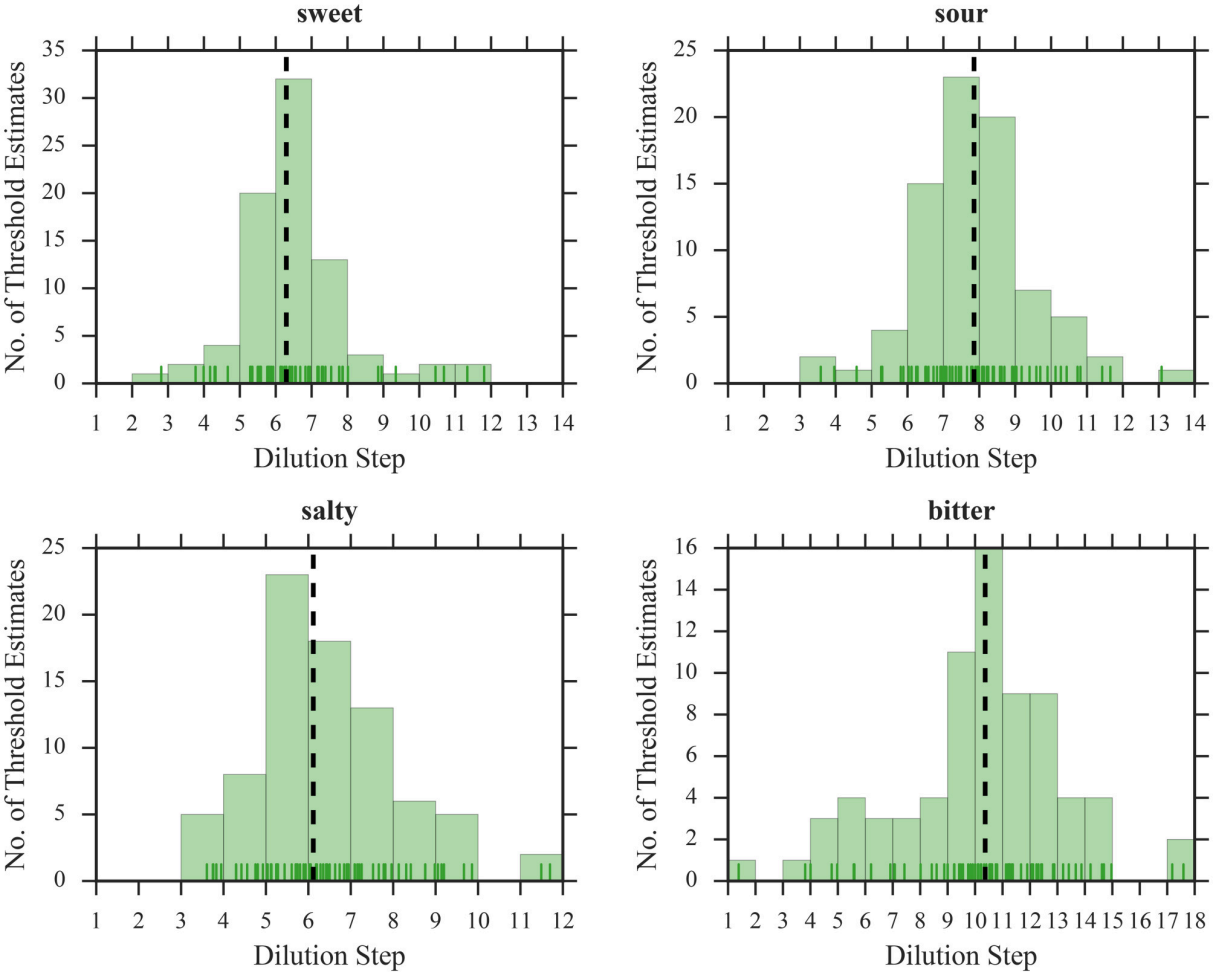
Distribution of threshold estimates for QUEST. Blue vertical lines depict individual participants; black dashed lines represent the medians.

To illustrate the suitability of the selected concentration ranges, we plotted histograms of all thresholds as a function of dilution steps (Figures 1, 2). The medians were located in the central regions of concentration ranges, and only 6 out of 608 thresholds were found in the “extreme” bins, indicating that the selected concentration ranges were in fact sufficient for most participants.

### 3.3. Mean thresholds

**Figure 3:**
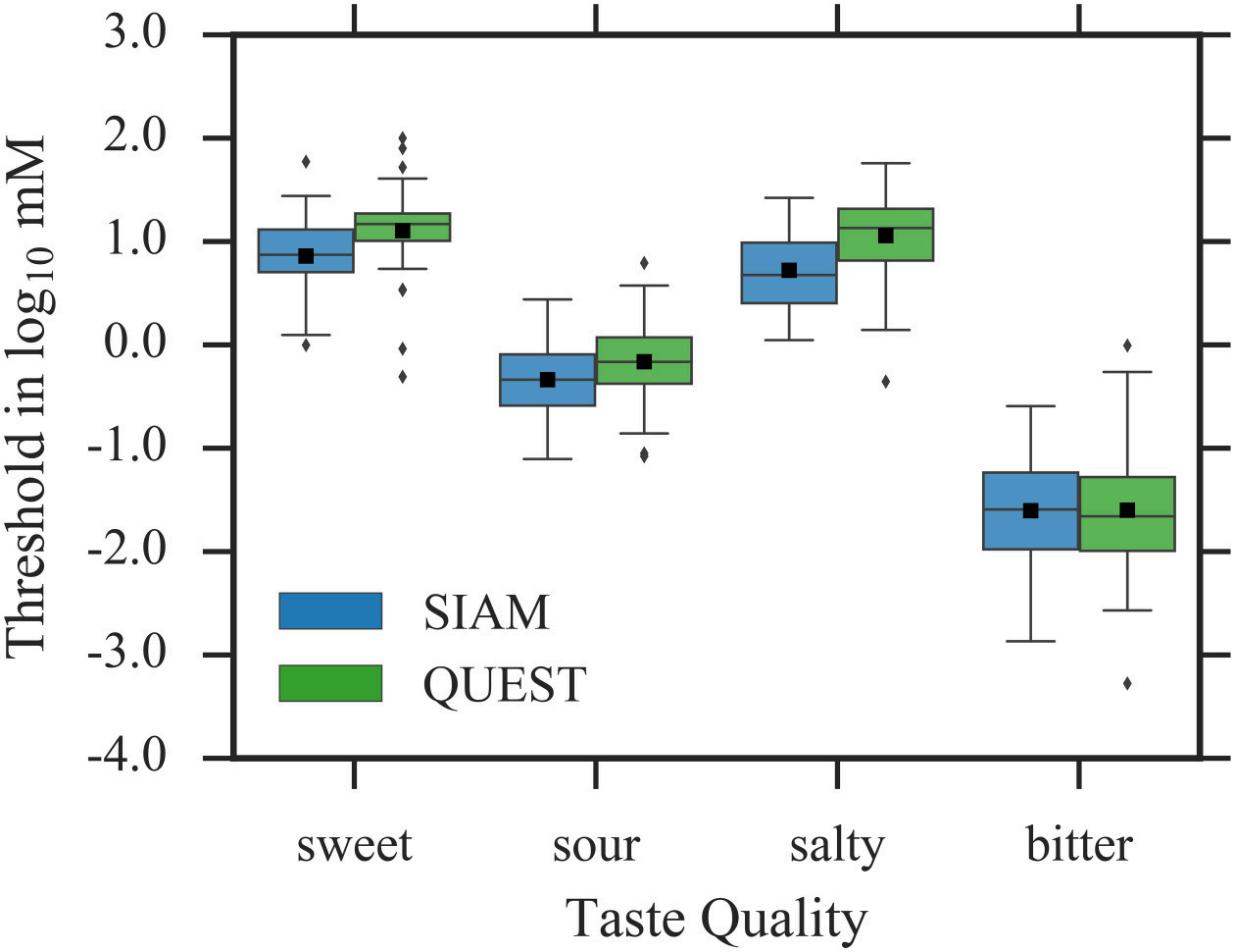
Threshold estimates, averaged across test and retest. Whiskers are 1.5 × interquartile range. Outliers are depicted as diamonds, and sample means as black squares.

The mean thresholds, averaged across test and retest, are plotted separately for SIAM and QUEST in Figure 3. As expected, mean thresholds for QUEST were significantly higher than for SIAM for sweet [*F*_(1, 117)_ = 12.44, *p* < 0.001], sour [*F*_(1, 113)_ = 12.34, *p* < 0.001], and salty [*F*_(1, 118)_ = 30.78, *p* < 0.001]; no difference between procedures was observed for bitter [*F*_(1, 109)_ = 0.06, *p* = 0.81].

### 3.4. Test-retest reliability

**Figure 4:**
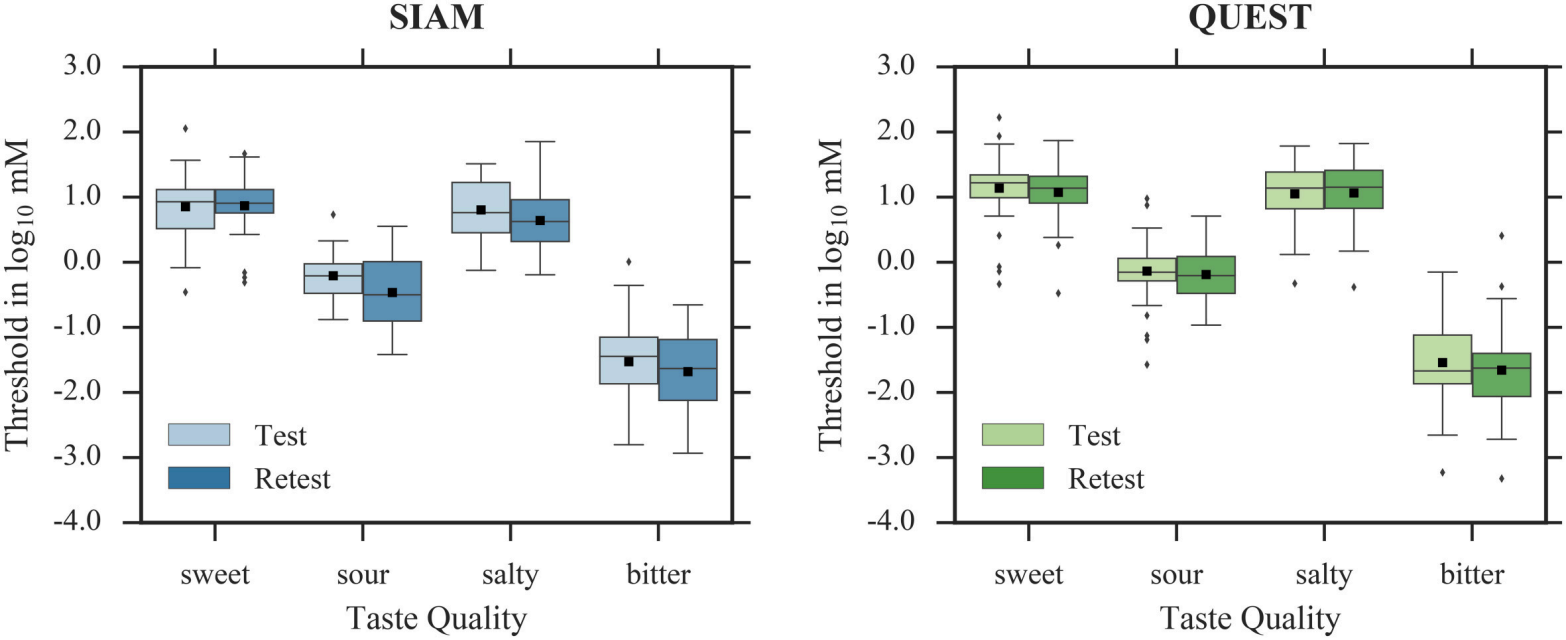
Threshold estimates in test and retest for SIAM (left) and QUEST (right). Whiskers are 1.5 × interquartile range. Outliers are depicted as diamonds, and sample means as black squares.

**Figure 5:**
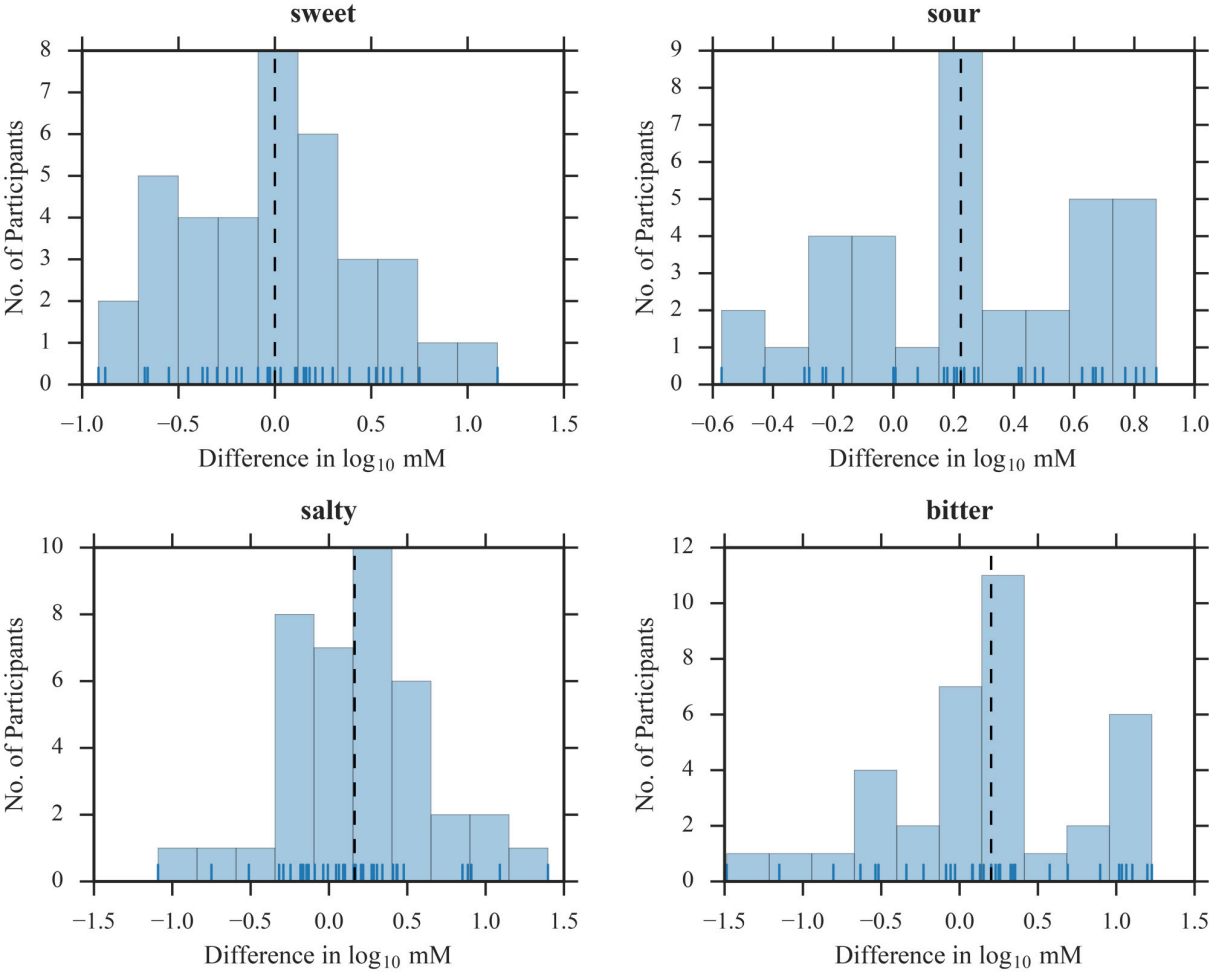
Distribution of threshold differences between test and retest for SIAM. Blue vertical lines depict individual participants; black dashed lines represent the medians.

**Figure 6:**
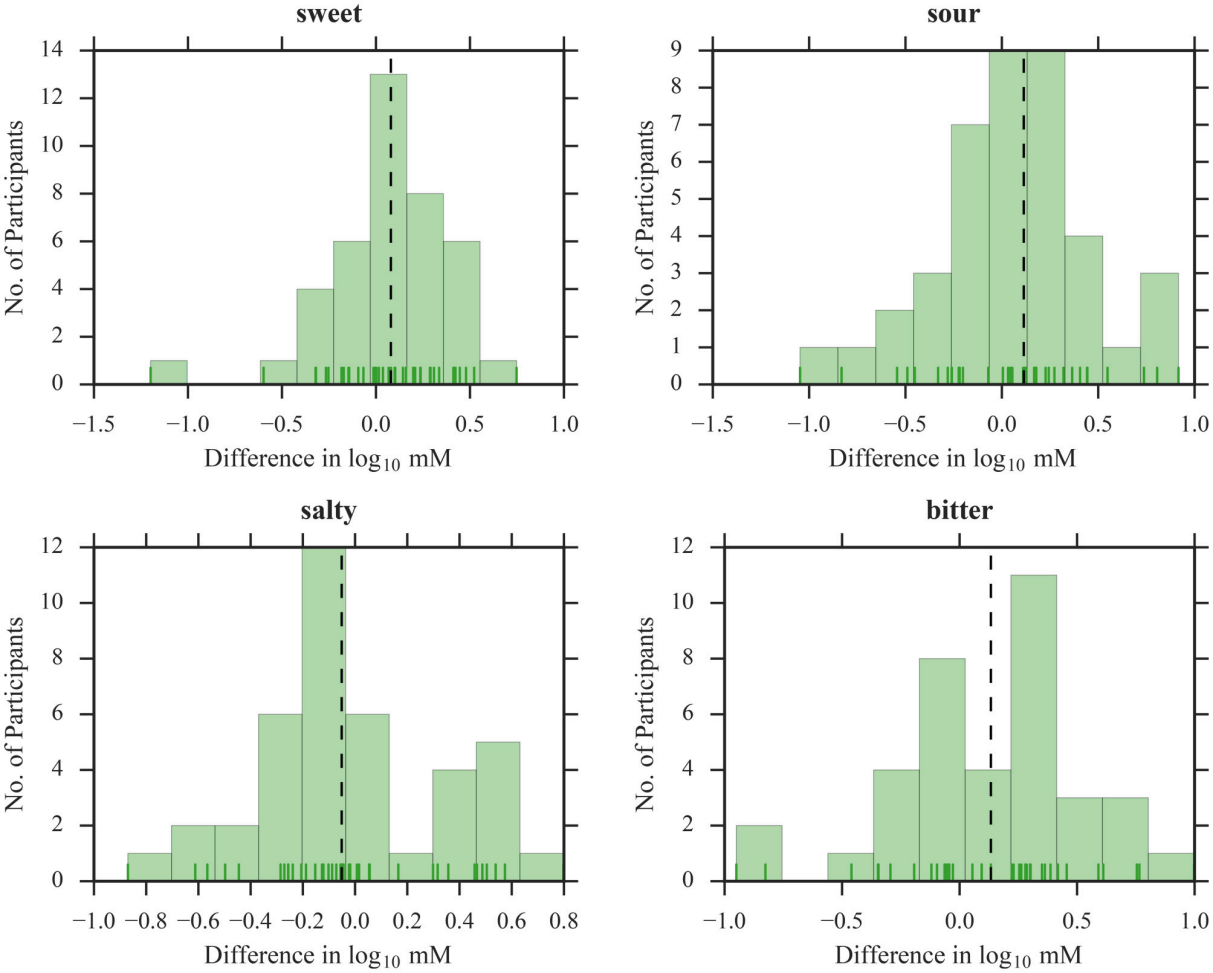
Distribution of threshold differences between test and retest for QUEST. Green vertical lines depict individual participants; black dashed lines represent the medians.

Thresholds for test and retest are shown in Figure 4; averaged thresholds, their respective standard deviations, and differences between test and retest are summarized in Table 3; and distributions of the absolute threshold differences between sessions are shown in Figures 5, 6. The average of the differences between test and retest threshold estimates was less than one concentration step for all taste qualities.

**Table 3:** Mean threshold estimates and respective standard deviations in test, retest, and the difference between both sessions.

**Method**	**Taste quality**	***N***	**Threshold in log**_**10 mM**_					
			**Test**		**Retest**		**Difference**	
			***Mean***	***SD***	***Mean***	***SD***	***Mean***	***SD***
SIAM	Sweet	37	0.852	0.490	0.867	0.443	−0.015	0.478
	Sour	35	−0.208	0.330	−0.465	0.545	0.257	0.393
	Salty	39	0.803	0.431	0.641	0.457	0.162	0.477
	Bitter	36	−1.528	0.669	−1.681	0.611	0.152	0.646
QUEST	Sweet	40	1.137	0.502	1.073	0.410	0.064	0.341
	Sour	40	−0.137	0.487	−0.189	0.401	0.053	0.408
	Salty	40	1.050	0.442	1.064	0.498	−0.014	0.355
	Bitter	37	−1.542	0.704	−1.657	0.697	0.116	0.407

**Figure 7:**
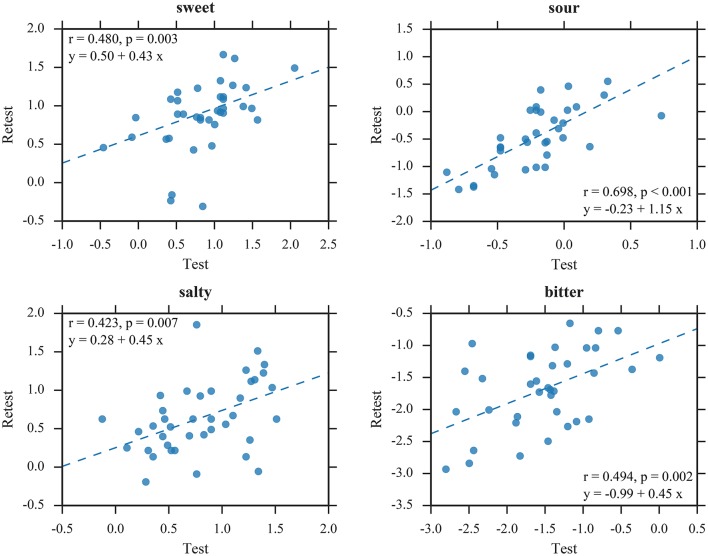
Test-retest correlations of SIAM thresholds. All values in log_10_mM.

**Figure 8:**
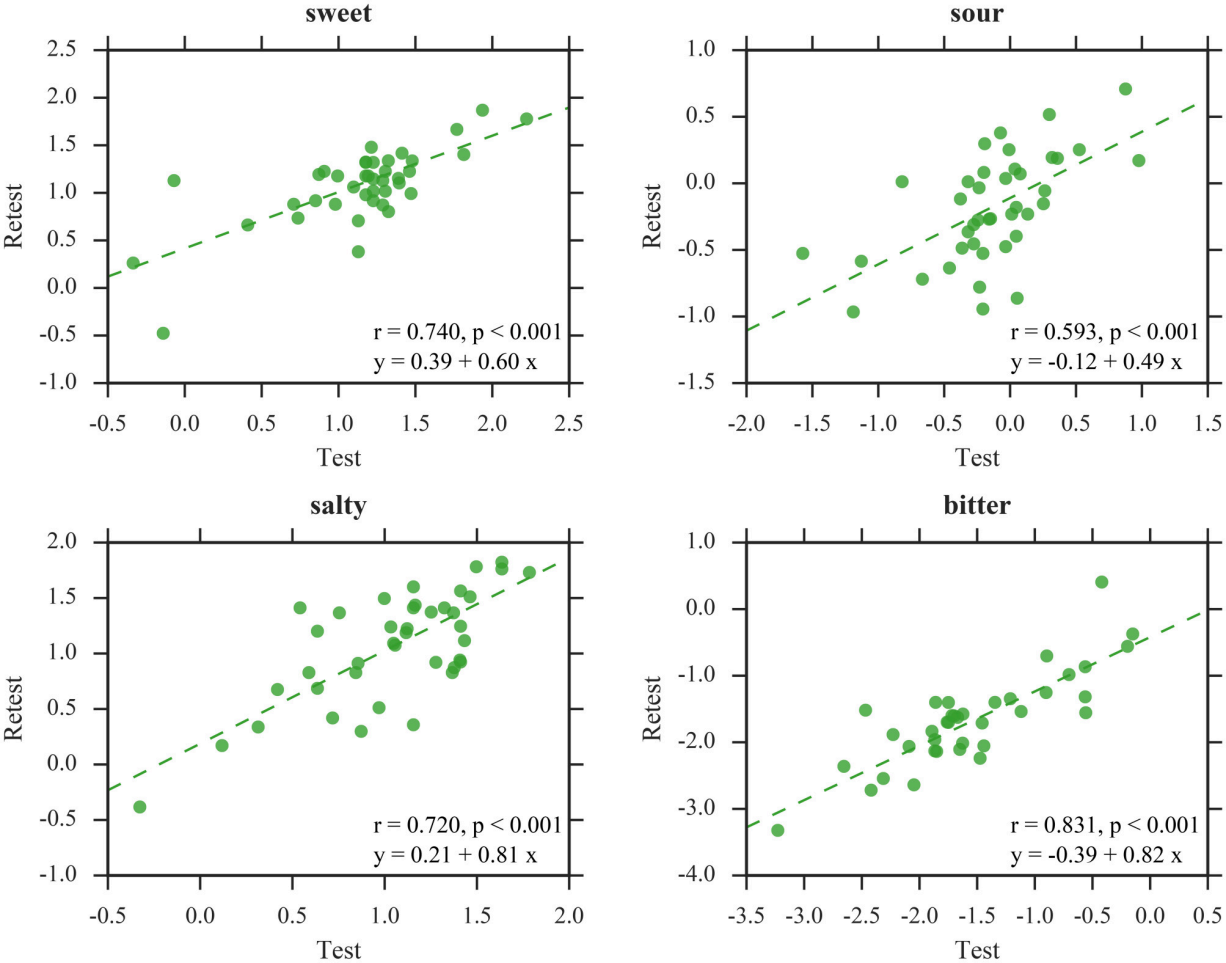
Test-retest correlations of QUEST thresholds. All values in log_10_mM.

The LMMs revealed no significant main effect of *session* for sweet [*F*_(1, 110)_ = 0.15, *p* = 0.70], salty [*F*_(1, 114)_ = 1.51, *p* = 0.22], and bitter [*F*_(1, 104)_ = 2.60, *p* = 0.11], indicating that test and retest yielded similar thresholds for SIAM and QUEST. However, thresholds were lower during retest compared to test for sour [*F*_(1, 106)_ = 7.35, *p* < 0.01]. Accordingly, SIAM thresholds did not differ significantly between sessions for sweet [*t*_(109)_ = 0.16, *p* = 0.88], salty [*t*_(112)_ = −1.89, *p* = 0.06], and bitter [*t*_(105)_ = −1.29, *p* = 0.20]; however, thresholds were lower during retest compared to test for sour [*t*_(103)_ = −3.08, *p* < 0.01]. The correlation analysis for SIAM thresholds (Figure 7) suggests mediocre test-retest reliabilities for sweet [*r*_(37)_ = 0.48], salty [*r*_(39)_ = 0.42], and bitter [*r*_(36)_ = 0.49] (all *p* < 0.01), and a high test-retest reliability for sour [*r*_(35)_ = 0.70, *p* < 0.001]. Similarly, QUEST thresholds did not differ significantly between sessions for any taste quality [sweet: *t*_(109)_ = −0.75, *p* = 0.48; sour: *t*_(103)_ = −0.67, *p* = 0.50; salty: *t*_(112)_ = 0.16, *p* = 0.87; bitter: *t*_(105)_ = −0.99, *p* = 0.32]. Test-retest correlations (Figure 8) were high for all taste qualities [sweet: *r*_(40)_ = 0.74, sour: *r*_(40)_ = 0.59, salty: *r*_(40)_ = 0.72, bitter: *r*_(37)_ = 0.83; all *p* < 0.001]. No significant interactions between *staircase type* and *session* were observed.

#### 3.4.1. Correlation SIAM—QUEST

**Figure 9:**
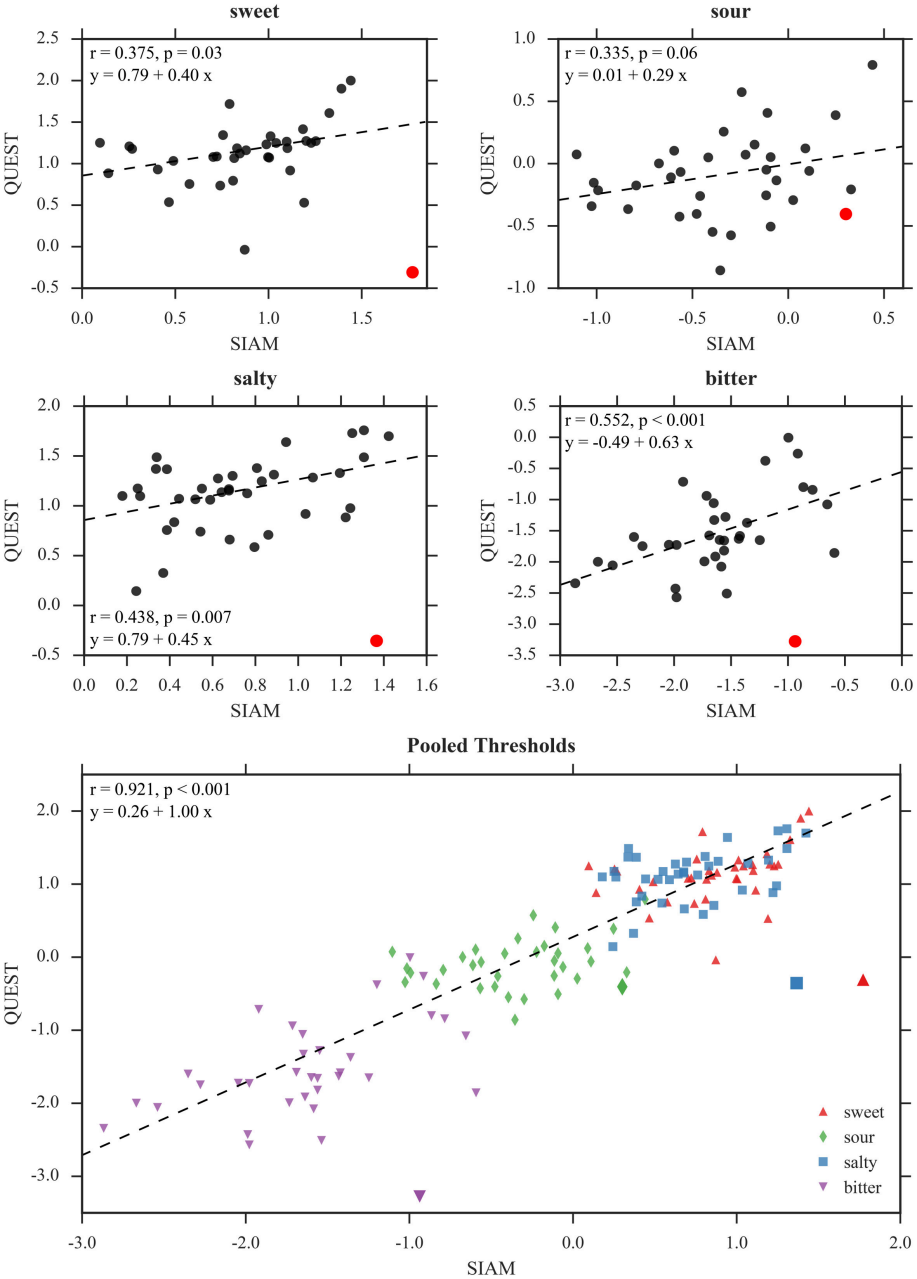
Correlations between mean threshold estimates of SIAM and QUEST. Top panels: Separate correlations calculated for each individual taste quality. The large red dots depicts the “outlier” that was excluded from the analyses presented here (but see text for coefficients with these data included). Bottom panel: Data pooled across all taste qualities. The “outlier” is depicted by larger markers than the other data points and was excluded from correlation analysis. All values in log_10_mM.

Linear relationships between the threshold estimates of individual taste qualities assessed with SIAM and QUEST were weak to moderate, and significant only for bitter [*r*_(34)_ = 0.40, *p* = 0.02], but non-significant for sweet [*r*_(36)_ = 0.07, *p* = 0.68], sour [*r*_(34)_ = 0.28, *p* = 0.12], and salty [*r*_(38)_ = 0.19, *p* = 0.26]. Through visual inspection, we discovered one participant (female, age: 54 yr, BMI: 30.9 kg m-2) with particularly inconsistent threshold estimates between methods for sweet, salty, and bitter. After excluding this “outlier participant” from correlation analysis, the correlations greatly increased and became significant for three of the four tastants [sweet: *r*_(35)_ = 0.38, *p* = 0.03; sour: *r*_(33)_ = 0.34, *p* = 0.06; salty: *r*_(37)_ = 0.44, *p* < 0.01; bitter: *r*_(33)_ = 0.55, *p* < 0.001; *p*-values not corrected for multiple testing]. See Figure 9 (top panels).

A joint plot of the mean QUEST and SIAM thresholds pooled across taste qualities is shown in Figure 9 (bottom) and provides an overall impression of the obtained threshold estimates. Correlation between the methods was very high [*r*_(142)_ = 0.88, *p* < 0.001 including all data points, and *r*_(138)_ = 0.92, *p* < 0.001 with the “outlier” removed; *p*-values not corrected for multiple testing]. However, this result can possibly be explained by mean differences across the pooled subsets alone (Almeida-de Macedo et al., 2013).

## 4. Discussion

The present study aimed to evaluate the applicability of two adaptive methods based on SIAM and QUEST for the time-efficient and reliable estimation of taste thresholds. The results show that both methods measure taste thresholds rapidly (within 6.5 min to 9.5 min) and reliably.

### 4.1. Test-retest reliability

Although the threshold procedures were specifically designed for a rapid estimation of taste thresholds, we observed test-retest reliabilities (*r* = 0.42 to *r* = 0.70 for SIAM and *r* = 0.59 to *r* = 0.83 for QUEST) that compare well with previous studies. The lower test-retest correlations for SIAM than QUEST in three of the four taste qualities may be linked to the susceptibility of SIAM to the number of trials (see Section 4.3). High test-retest correlations have been reported for citric acid (*r* = 0.77) and sodium chloride (*r* = 0.70) using a modified Harris-Kalmus procedure (Wise and Breslin, 2013). Similar results were obtained for sucrose with an inter-session interval of 1 day (*r* = 0.86) and 1 week (*r* = 0.76) via a forced-choice staircase procedure (Mattes, 1988). Using the three-drop method, Mueller et al. (2003) found relatively diverse test-retest correlations for sucrose (*r* = 0.50), citric acid (*r* = 0.36), sodium chloride (*r* = 0.37), and quinine hydrochloride (*r* = 0.61), and similar results for taste strips (sucrose: *r* = 0.43, citric acid: *r* = 0.40, sodium chloride: *r* = 0.34, quinine hydrochloride: *r* = 0.56). The variability of of these results can partly be explained by different test-retest intervals. Test-retest reliability of sucrose (Mattes, 1988) and sodium chloride thresholds (Linschoten et al., 2001) has been found to decrease with increasing intervals between sessions. In the present study, participants completed both sessions within less than 2 days on average. Whether the correlation changes with longer inter-session intervals remains to be investigated. Notably, all aforementioned procedures either place a higher demand on participants' cognitive and mnemonic functions, require more testing time, or produce much less fine-grained threshold estimates than the SIAM and QUEST methods presented here.

### 4.2. Response bias

A common challenge when estimating sensitivity thresholds is to differentiate between actual sensory sensitivity and cognitive processes that influence task performance. The tendency to either respond *Yes* or *No* to a given stimulus concentration, the so-called *response bias* or *response criterion*, must be taken into consideration and controlled for. This criterion can be estimated by measuring not only hits, but also false alarms produced by a participant. For that reason, SIAM introduces blank stimuli on 50% of all trials. QUEST does not allow for an estimation or correction of response bias in a yes-no paradigm, and hence does not belong to the category of “objective” methods according to Klein (2001). Still, we employed this method and instructed participants to apply a “conservative” response criterion. The higher thresholds in comparison to SIAM in three of the four taste qualities suggest that participants did in fact adhere to a conservative criterion, or at least did not consistently produce false alarms; in fact, we rarely observed *Yes* responses to the weakest stimuli during the QUEST procedure (which consequently led to exclusion of the respective data sets), supporting our choice of a PF with the lower asymptote close to zero. The good test-retest reliability for QUEST indicates that the criterion remained stable across sessions although participants could not recalibrate their response criterion in the absence of trial-by-trial feedback. However, we cannot exclude that the granularity of the dilutions steps contributed to these findings.

Peripheral effects pose another challenge to threshold estimations. The use of water as stimulus and rinse may induce a taste perception alone and hence a false alarm due to removal of chemical compounds from the tongue surface (Galindo-Cuspinera et al., 2006). Additionally, water rinse has been found to affect sodium chloride sensitivity (Weiffenbach et al., 1983). Also, we cannot exclude carry-over effects between trials and potentially even between taste qualities although ISIs were long and participants were free to use as much rinsing water as desired. The latter may have served as a source of between-subject variability, though, because some participants rinsed with smaller amounts of water, while others used larger quantities to remove (after)tastes between trials.

### 4.3. Efficiency

Because SIAM's stimulus selection is based on the previous response alone, the procedure sometimes requires a substantial number of trials to converge to the threshold region, especially if participants produced a false alarm early in the experiment. QUEST, on the other hand, was more robust to inconsistent responses due to its Bayesian approach, which incorporates prior knowledge about the *entire* experimental run. On average, QUEST required less than half the trials compared to SIAM to successfully estimate a threshold with an overall superior test-retest reliability.

SIAM has been previously used to measure sucrose sensitivity with whole-mouth stimulation, and the authors observed threshold values in a similar range as reported here (0.81 mm to 0.89 mm; Hautus et al., 2010; Shepherd et al., 2011). While SIAM thresholds obtained from sessions of 60 trials were comparable to those from a 2-IFC task (Shepherd et al., 2011), significant differences were observed between SIAM and 2-IFC in sessions with only 30 SIAM trials (Hautus et al., 2010). In the latter case, some participants showed large variability across sessions. We derived test-retest correlations from the data provided by Hautus et al. (2010, Table 1) and found low correlation coefficients [Session 1–2: *r*_(15)_ = 0.13, *p* = 0.64; Session 2–3: *r*_(15)_ = 0.21, *p* = 0.45; Session 1–3: *r*_(15)_ = 0.12, *p* = 0.68; *p*-values not corrected for multiple testing]. These results suggest that 30 SIAM trials are insufficient to reliably and accurately estimate taste thresholds. Although the test-retest correlations for SIAM presented here are larger than those in the data of Hautus et al. (2010), SIAM procedures with more than 30 trials likely exhibit more robust results. Accordingly, within-participant inconsistency of the SIAM thresholds may have led to the weak correlation between SIAM and QUEST thresholds in the present study.

### 4.4. Application

While stimuli for the non-chemical senses can often be generated on-line with computer systems, allowing for very quick and fine-grained adjustments e.g., of stimulus contrasts in vision research, taste stimuli have to be produced prior to the experimental session by mixing a series of different aqueous solutions for any given tastant. Tastants are often applied with pipettes (e.g., Kroeze and Bartoshuk, 1985), delivered in cups (e.g., Wise and Breslin, 2013), or via automated syringe pump systems, mostly in electrophysiological or neuroimaging studies (see e.g., Small, 2010; Ohla et al., 2011). Previous attempts to develop portable delivery options that do not require a laboratory environment have led to the implementation of filter paper strips soaked with tastants (“taste strips”; Mueller et al., 2003; Landis et al., 2009), edible taste strips (Smutzer et al., 2008), and taste tablets (Ahne et al., 2000). Taste strips were specifically developed for the assessment of gustatory function in clinical context and at bedside. The method is easy to apply, quick, and portable, as it does not require preparation of liquid tastants. It has been optimized for the assessment of overall taste function and classification of normogeusic, hypogeusic, and ageusic patients. While a comparative interpretation of existing findings is exacerbated by methodological differences, particularly in tastant application, threshold algorithms, and tasks between studies, our results suggest that good test-retest reliability can be achieved in a time-efficient manner with few trials using liquid tastants. In the absence of normative data, however, an interpretation of the results of the present and of previous studies is limited with reference to data from the same or similar studies.

## 5. Conclusion

In the present study, we combined easy-to-use and portable spray bottles with adaptive algorithms to allow for a quick and reliable, yet fine-grained taste sensitivity estimation. The presented dilution series were sufficiently wide to allow identification of thresholds in most individuals. The adapted QUEST procedure yielded better test-retest reliability than SIAM in for most taste qualities and has already been used to differentiate between groups of participants in another study (Hardikar et al., 2016). SIAM performance could likely be improved by larger numbers of trials, which, however, would also increase the required testing time. Overall, we propose that the QUEST procedure is ideal for large cohort studies and clinical applications likewise, and may, therefore, serve as a complementary diagnostic tool once normative data are provided. In order to facilitate this process, the source code of the presented algorithms will be provided upon request.

## Author contributions

RH and KO conceived the experiments, RH conducted the experiments and analyzed the data, RH and KO interpreted the data and wrote the manuscript.

### Conflict of interest statement

The authors declare that the research was conducted in the absence of any commercial or financial relationships that could be construed as a potential conflict of interest.
